# Microbial quality assessment of minimally processed pineapple using GCMS and FTIR in tandem with chemometrics

**DOI:** 10.1038/s41598-020-62895-y

**Published:** 2020-04-10

**Authors:** Vanshika Adiani, Sumit Gupta, Prasad S. Variyar

**Affiliations:** 1 Food Technology Division, Bhabha Atomic Research Centre, Mumbai, India; 2 Homi Bhabha National Institute, Anushakti Nagar, Mumbai, India

**Keywords:** Gas chromatography, Solid-phase microextraction

## Abstract

Microbial quality is the critical parameter determining the safety of refrigerated perishables. Traditional methods used for assessing microbial quality are time consuming and labour intensive. Thus rapid, non-destructive methods that can accurately predict microbial status is warranted. Models using partial least square regression (PLS-R) from chemical finger prints of minimally processed pineapple during storage obtained by Headspace Solid Phase Microextraction Gas Chromatography Mass Spectrometry (HS-SPME-GCMS), Fourier Transform Infrared (FTIR) spectroscopy and their data fusion are developed. Models built using FTIR data demonstrated good prediction for unknown samples kept under non-isothermal conditions. FTIR based models could predict 87 and 80% samples within ±1 log CFU/g for TVC and Y&M, respectively. Analysis of PLS-R results suggested the production of alcohols and esters with utilization of sugars due to microbial spoilage.

## Introduction

Monitoring the microbial quality is a critical factor in assuring safety and quality of food products in modern supply chains^1^. Conventional technique such as standard microbial plate count technique requires 48–72 h to evaluate the microbial quality, which is not suitable for fast deteriorating products with a short shelf life. Several non-destructive techniques have been proposed that offers online rapid monitoring of product microbial quality when integrated with multivariate chemometric tools such as imaging^2^ in meat samples, spectroscopic^3–5^ in beef, jackfruit and pineapple, hyperspectral imaging^6,7^, e-nose^8^ in several agro-food products and acoustic^9^ for fruit juices. Although, these techniques have offered reduced time of analysis along with informative results, but their suitability has been demonstrated in microbial quality assessment on subset of same batch of sample used for training chemometric models. None of the research work conducted has demonstrated the utility of the developed models for prediction of completely blind unknown set of samples. Moreover, as quality assurance deals with food safety it becomes crucial to test the reliability and efficiency of prediction model developed with samples from unknown batch. Building models that have the capability to predict microbial quality of unknown samples with minimum error in least possible time will lead to faster adoption of these techniques in industry.

Both, microbial growth and enzymatic activity during spoilage of food products results in several chemical changes that lead to product deterioration. Changes in volatile constituents during the product deterioration can be rapidly monitored by Headspace Solid Phase Microextraction (HS-SPME) in combination with Gas chromatography mass spectrometry (GCMS). The other non-volatile biochemical changes can be monitored by Fourier transform infrared spectroscopy (FTIR). Partial Least Square Regression (PLS-R) is the most popular tool utilized for quantitative regression analysis applied in the food industry^10^. This technique is most suitable when number of variables is greater than sample size and when they are collinear.

In addition, recent studies employing data fusion methodologies using more than one analytical technique have offered a practical way to lower error of prediction by 5–20% unlike utilizing data from single analytical equipment for chemometric modeling^10^. Several approaches of data fusion methods have been employed for quality monitoring in terms of authentication, discrimination, adulteration and prediction in food samples that has reduced the error of performance^11–13^. Several such reports have led to interest in exploring data fusion methodologies that will decrease the uncertainty of individual result and enable superior performance in prediction^12^. However, such methods have not been applied for microbial quality monitoring. Thus, the overall objective of this work is to develop rapid method for microbial quality estimation of minimally processed pineapple at different storage temperatures using GCMS and FTIR in combination with chemometric tools such as PLS-R. Performance of these models will be tested for its utility in industry with completely unknown set kept under non-isothermal condition of storage to simulate market conditions. Data fusion of these two techniques will also be attempted to increase the efficiency of performance of the models which may lead to better prediction of microbial counts.

## Results and Discussion

### Microbial analysis

**Figure 1:**
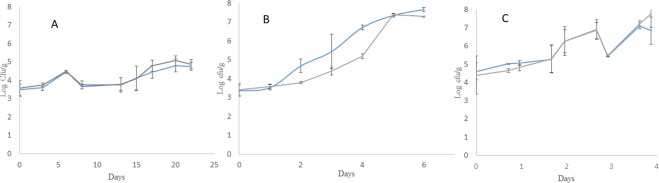
Total viable count () and Yeast & Mould () count of minimally processed stored pineapple. (**A**) Samples stored at 4 °C (**B**) Samples stored at 10 °C and (**C**) unknown set stored at non-isothermal conditions with periodic 24 h cycle of 16 h at 10 °C and 4 h at 15 °C then finally for 4 h at 20 °C.

The initial microbial counts (day 0) were 3.49 ± 0.31 and 3.56 ± 0.42 Log CFU/g in case of total viable count (TVC) and yeast and mould count (Y&M), respectively. Samples stored at 4 °C demonstrated a marginal increase in the microbial counts to 4.77 ± 0.21 and 4.89 ± 0.24 Log CFU/g for TVC and Y&M, respectively by the end of the storage period at 22 days (Fig. 1A). However, beyond 10 days physiological deterioration resulting from browning and water loss was observed. Previous studies on cut pineapple^14,15^ supports this observation. In contrast, samples stored at 10 °C demonstrated a rapid increase in TVC and Y&M with counts reaching to 7.92 ± 0.32 and 7.91 ± 0.15 Log CFU/g, respectively by the end of the storage period of 7 days (Fig. 1B). An enhanced TVC and Y&M counts was also observed for sample set stored under non-isothermal conditions with counts reaching to 6.85 ± 0.74 Log CFU/g and 7.76 ± 0.68 Log CFU/g on day 4 (Fig. 1C). Thus minimally processed products have short shelf life of few days primarily due to microbial growth necessitating development of rapid methods of assessment of microbial quality.

### HS/SPME-GCMS analysis

**Figure 2:**
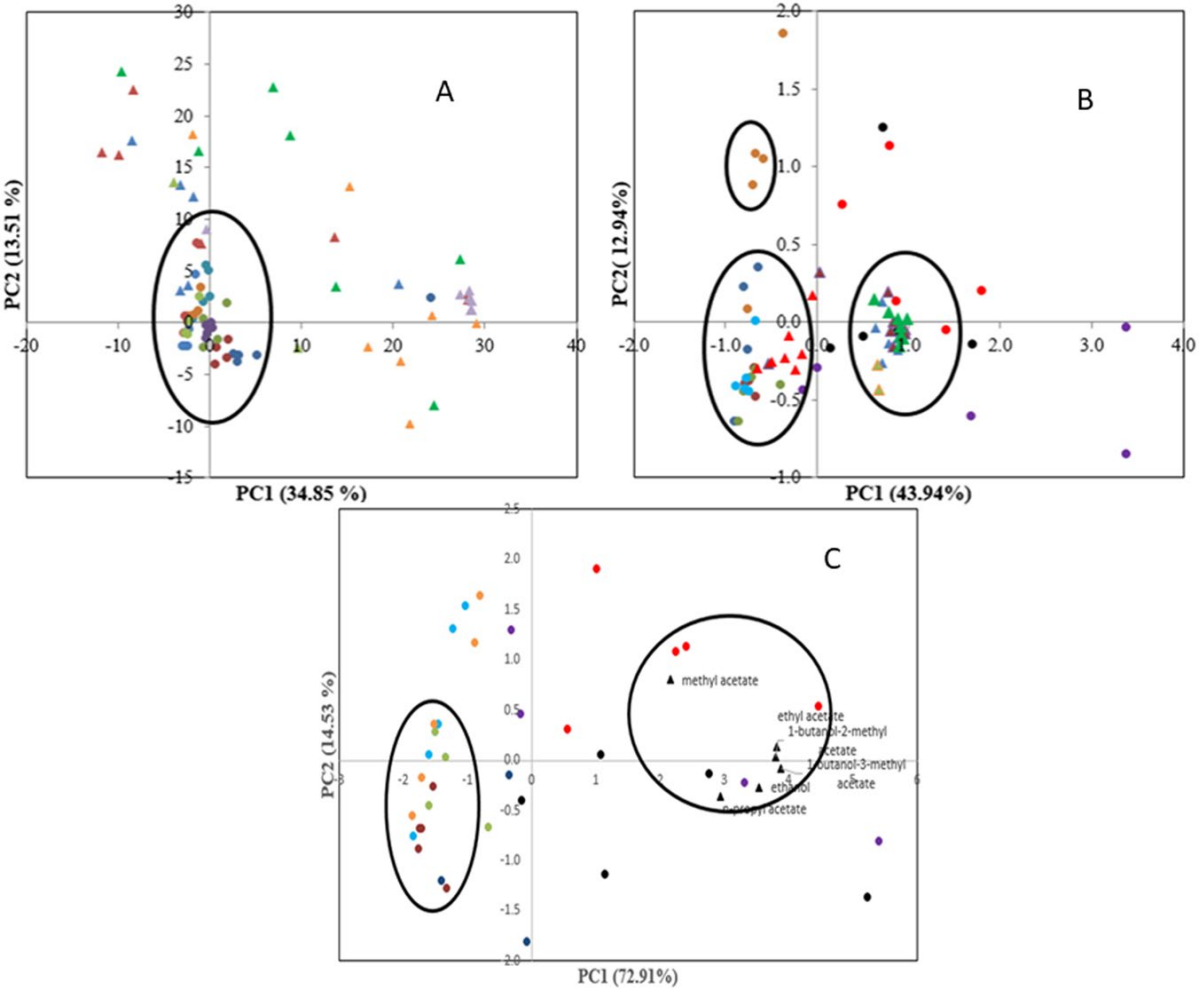
Principal component analysis of minimally processed stored pineapple A) GCMS data (whole volatile profile) of samples stored at 4 °C, () Day 0 ()Day 3 ()Day 6 () Day 8 () Day 13 () Day 15 () Day 17 () Day 20 () Day 22. B) GCMS data (whole volatile profile) of samples stored at 10 °C  alcohol,  acetates,  methyl esters,  ketone,  ethyl esters and C) GCMS data (with 6 volatiles) of samples stored at 10 °C. () Day 0 ()Day 1 ()Day 2 () Day 3 () Day 4 () Day 5 () Day 6 () Day 7.

Table S1 shows the concentration of the 44 volatile compounds that were obtained at the beginning and end of storage period. No segregation was observed in principal component score plots (Fig. 2A) for samples stored at 4 °C suggesting no significant changes in volatile constituents during storage (Table S1, Fig. 2A). Previous study on the volatile profile of stored minimally processed pineapple at different temperatures also observed no significant changes in the volatile compounds at 4 °C when compared to samples stored at higher temperature^16^. Further, as only a marginal increase in microbial counts was observed in samples stored at 4 °C, quality assessment using chemometrics tools was not further carried out for these samples.

Score plot for 10 °C stored samples showed segregation in three different groups (Fig. 2B). First group had samples stored up to 3 days, day 4 samples belongs to the second group, while last group comprised of samples stored from day 5 to day 7 (Fig. 2B). Methyl esters were found to be positively correlated to the first group. These esters are reported to impart characteristic fresh ripe aroma of pineapple^17^. Positive correlation of alcohols, ethyl esters, acetates and ketones was observed with the third group (day 5 to 7).

Correlation analysis revealed 9 volatile compounds namely ethanol, methyl acetate, ethyl acetate, n-propyl acetate, 3-methyl-1-butanol, 1-butanol-3-methyl acetate, 1-butanol-2-methyl acetate, 2-heptanone, and 2-phenyl ethyl acetate were positively correlated (R > 0.5) with microbial counts. A similar correlation was also observed by other researchers^17,18^. Production of ethanol can be attributed to *Pseudomonads* and crab free positive yeast under aerobic conditions^18,19^, while formation of acetate esters coincided with the onset of fermentation during storage by yeast activity^20–22^. Previous studies also demonstrated strong correlation of microbial count (>0.6) with ethanol, ethyl acetate and 3-methyl-1-butanol^4,23^. Some of the volatile compounds such as 3-methyl-1-butanol, 2-phenyl ethyl acetate and 2-heptanone were not detected in all the stored pineapple samples.

Score plot obtained from PCA analysis based on microbial generated volatiles (ethanol, methyl acetate, ethyl acetate, n-propyl acetate, 1-butanol-2-methyl acetate, 1-butanol-3-methyl acetate), revealed clear segregation of samples stored up to 3 days from samples stored for longer periods (day 5 to 8) (Fig. 2C). Positive correlation of these volatiles with samples stored for longer period (day 5 to 8) was also observed. Therefore, these six volatiles were employed for building chemometric models to predict the microbial quality of minimally processed pineapples.

### FTIR analysis

**Figure 3:**
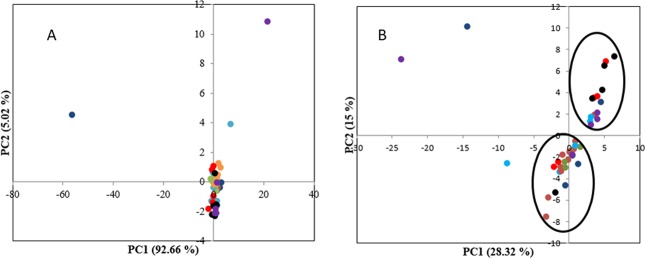
Principal component analysis of minimally processed stored pineapple samples at 10 °C (**A**) FTIR spectral data (**B**) FTIR first derivative data () Day 0 () Day 1 () Day 2 () Day 3 () Day 4 () Day 5 () Day 6 () Day 7.

FTIR spectra in the fingerprint region range of 1000–2000 cm^−1^ that carried maximum information was utilised for microbial quality assessment^5^. The representative FTIR profile is provided in Supplementary Information (Figs. S2 and S3). A major peak at 1638 cm^−1^ corresponds to carbonyl stretch of conjugate ketone or quinone^24^, the peak at 1418 and 1364 cm^−1^ corresponds to O-C-H, C-C-H, C-O-H bending and deformation of carbohydrates^25^ while 1258 cm^−1^ represents -C = O acid stretching^26^ and 1105 cm^−1^ and 1054 cm^−1^ to C-O & C-H stretching vibration of sugars^27^. PCA analysis of FTIR spectral data revealed that the first two PCs explained 97.68% variance, however no segregation of samples based on storage time was observed (Fig. 3A). First derivative function was therefore employed to resolve overlapping peaks. This resulted in first two PCs accounting for 43.43% variance and segregation of samples based on storage time was also noted. Samples stored up to 4 days constituted one group and was located on negative axis of PC2 whereas samples from 5 to 7 days formed another group located on positive side of PC1 and PC2 (Fig. 3B). Thus use of first derivative function could reveal the difference in the stored samples. Both FTIR spectral data and FTIR first derivative data were utilized for building models for assessment of microbial quality. The model performance was then evaluated for unknown samples stored under non-isothermal conditions.

### GCMS and FTIR based quantitative prediction

PLS-R models were built for TVC and Y&M counts by employing instrumental data (details as described in methodology) as independent variables and microbial counts (Log_10_ CFU/g) of pineapple samples (stored at 10 °C) as dependent variable. While building PLS-R models, selection of number of latent variables (LV) for model building is an important step. Fewer number of LVs results in insufficient model while too many LVs lead to overfitting with calibration dataset. Both these cases could possibly result in poor performance of models for prediction data^28^. In the present study, number of LVs to be used for PLS-R model building was based on RMSECV (Root mean square error of cross validation) for the calibration data carried out with leave one out approach. Plots of RMSECV versus LVs for different forms of data are provided in Fig. S4 (Supplementary information). Final regression models were prepared using that number of latent variables which on further increase resulted in either constant or increased RMSECV. The number of latent variables finally selected for building models and their corresponding RMSECV values are shown in Table 1. Models prepared were evaluated for their suitability by analysing their performance for different set of samples previously unused for training. Moreover, samples used for prediction set were stored under non-isothermal conditions (conditions described in methodology) to simulate actual market conditions. The accuracy and bias factors, SEP and R^2^ predicted obtained for prediction samples are shown in Table 1.

**Table 1:** Performance parameters of models generated by PLS-R using different forms of data.

		LV	RMSECv	R^2^_cal_	R^2^_pred_	SEP	A_*f*_	B_*f*_	Within ±1 Log cfu/g (%)
TVC	GCMS	2	1.18	0.573	0.11	2.05	1.25	1.21	53
	FTIR spectral	4	1.06	0.70	0.51	0.70	1.10	0.96	87
	FTIR first derivative	2	1.36	0.877	0.61	0.69	1.08	0.94	87
	LL-GCMS-FTIR SPECTRAL	5	1.16	0.72	0.35	2.74	1.34	1.27	37
	LL-GCMS-FTIR first derivative	1	1.47	0.42	0.54	0.78	1.11	1.01	87
	IL-GCMS-FTIR spectral	3	1.36	0.39	0.13	1.41	1.23	1.20	40
	IL-GCMS-FTIR first derivative	1	1.49	0.42	0.55	0.76	1.12	1.06	87
Y&M	GCMS	1	0.77	0.786	0.20	1.44	1.20	1.01	53
	FTIR spectral	4	1.23	0.82	0.61	0.69	1.11	0.98	80
	FTIR first derivative	2	1.20	0.581	0.63	0.95	1.15	0.89	74
	LL-GCMS-FTIR Spectral	5	0.66	0.82	0.41	2.36	1.32	1.26	40
	LL-GCMS-FTIR first derivative	1	0.95	0.73	0.56	0.75	1.13	1.08	80
	IL-GCMS-FTIR spectral	3	0.98	0.73	0.25	1.43	1.24	1.21	40
	IL-GCMS-FTIR first derivative	1	1.10	0.73	0.57	0.81	1.11	0.98	74

R^2^-Correlation coefficient, SEP-Standard error of prediction, A_*f***–**_ accuracy factor, B_*f*_ – bias factor, LL – low level, IL – Intermediate level, LV- Latent variable.

**Figure 4:**
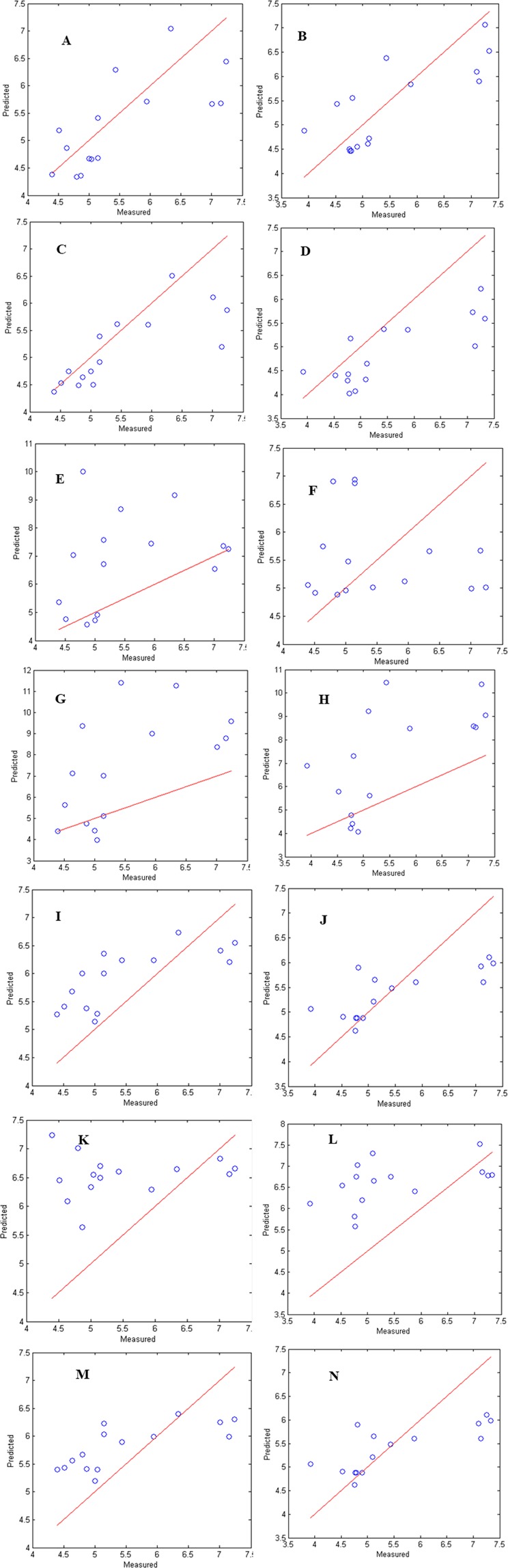
Comparison of total viable counts (TVC) and Yeast & Mold (Y&M) counts predicted using PLS model against experimentally observed values for prediction set stored under non-isothermal condition from FTIR spectral data (**A**,**B**); First derivative data (**C**,**D**); GCMS data, (**E**,**F**); LL-GCMS-FTIR spectral data (**G**,**H**); LL-GCMS-FTIR first derivative data (**I**,**J**); IL-GCMS-FTIR spectral data (**K**,**L**); IL-GCMS-FTIR first derivative data (**M**,**N**). Models build for TVC (**A**,**C**,**E**,**G**,**I**,**K**) on the left side of figure; Y&M (**B**,**D**,**F**,**H**,**J**,**L**,**M**) on the right side of figure. (**Solid line:** line of equity i.e. when predicted and actual values are hypothetical assumed to be same).

FTIR spectral data and first derivative data were utilized for building models for TVC and Y&M. Models built with FTIR spectral data had R^2^_pred_ of 0.51 for TVC and 0.61 for Y&M, respectively. Prediction done for unknown set using these models resulted in SEP of 0.70 and 0.69 with corresponding A_*f*_ values of 10 and 11% for TVC and Y&M, respectively (Table 1; Fig. 4A,B). FTIR first derivative data demonstrated SEP values of 0.69 and 0.95 with an A_*f*_ of 8 and 15% for TVC and Y&M, respectively. Moreover, FTIR first derivative data had a better R^2^_pred_ values of 0.61and 0.63 for TVC and Y&M, respectively. Results of prediction with FTIR first derivative based models are also depicted in Fig. 4C,D. Thus, FTIR first derivative data is reported to perform better when compared to raw FTIR spectra due to resolution of overlapping peaks^29,30^. In case of FTIR data, variable importance projection (VIP) scores >2 were obtained for peak at 1039, 1078 and 1105 cm^−1^ wavenumber. Peak at 1039 cm^−1^ corresponds to C-O and C-H stretching of sugars such as glucose and sucrose which was found to be negatively correlated with TVC and Y&M counts. Peaks at 1078 and 1105 cm^−1^ were positively correlated and corresponds to OH deformation of secondary and tertiary alcohols. Thus microbial activity leads to utilization of sugar and formation of alcohols and esters, these results are in agreement with previous studies^19,23,31^.

The models built using GCMS data demonstrated R^2^_pred_ value of 0.11 and 0.20 for TVC and Y&M, respectively indicating extremely poor performance for the prediction set. Further, the performance parameter such as SEP, A_*f*_ and B_*f*_ for TVC was 2.05, 25% and 1.21, respectively signifying a large deviation of 25% between actual and predicted values with considerable over prediction. Model performance for Y&M prediction were also low with values of SEP, A_*f*_ and B_*f*_ being 1.44, 20% and 1.01, respectively thereby suggesting higher deviation of 20% in prediction values with no systematic bias in prediction (Fig. 4E,F). The current attempts to predict the microbial counts for unknown samples stored under non-isothermal conditions using GCMS was not successful with high SEP values and large deviations in accuracy factor and low R^2^ values.

Using GCMS based models only 53% of both TVC and Y&M counts were predicted within ±1 log cfu/g. On the other hand FTIR spectral data and FTIR first derivative data were able to predict 87% TVC counts and 80 and 74% of Y&M counts within ±1 log cfu/g, respectively. Results obtained suggest that FTIR based models gave better results when compared to GCMS. For the GCMS prediction models, the VIP scores obtained for variables in PLS-R, it was observed that microbial generated volatiles ethyl acetate, 1-butanol 2-methyl acetate and 1-butanol 3-methyl acetate with variable scores >2 was obtained, suggesting strong correlation’s with TVC and Y&M.

Despite the fact that the models were tested with the samples belonging to different batch kept under non-isothermal conditions unlike the other studies reported in literature^3–5,32,33^, employing single technique (FTIR) more than 80% correct prediction within ±1 log cfu/g could be observed. Nevertheless, to further improve prediction performance and in order to reduce error, data fusion approaches were attempted on complementary techniques such as GCMS and FT-IR. While GCMS evaluates the off-odours produced due to microbial activity, FTIR provides fingerprint of non-volatile chemical changes occurring during food storage.

### Data fusion performance

Low level (LL) and intermediate level fusion of GCMS data (6 volatiles) individually with FTIR spectral data and first derivative data (147 variables each) was performed. LL-GCMS-FTIR spectral data showed a poor performance with R^2^_pred_ of 0.35 and 0.41 for TVC and Y&M, respectively with a very high SEP of 2.74 and 2.36. The developed models also showed a high average deviation in predicted samples for TVC and Y&M of 34 and 32%_,_ respectively with high B_*f*_ indicating over prediction of all the models developed (Fig. 4G,H, Table 1). In comparison to LL-GCMS-FTIR spectral data, fusion of LL-GCMS-FTIR first derivative data gave a better performance with R^2^_pred_ value of 0.56 and 0.54 for TVC and Y&M. This model also showed low SEP values of 0.78 and 0.75, along with low A_*f*_ of 11 and 13% for TVC and Y&M, respectively with a slight over prediction with B_*f*_ of 1.01 and 1.08, for TVC and Y&M, respectively (Fig. 4I,J).

Intermediate level (IL) data fusion was also attempted from the PC scores of the concatenated data obtained from low level fusion. The number of PC’s selected for IL-GCMS-FTIR spectral data and IL-GCMS-FTIR first derivative data were 4 and 27, respectively that explained 95% cumulative variability of the data. Similar to LL data fusion, low prediction performance was attained in case of IL GCMS-FTIR spectral data as well with high SEP of 1.41 and 1.43, respectively (Fig. 4K,L). IL GCMS-FTIR first derivative data showed better performance with SEP for 0.76 and 0.81 for TVC and Y&M, respectively (Fig. 4M,N).

Thus, to sum up single technique models built with FTIR first derivative data demonstrated better prediction performance than other models (Table 1). In data fusion, models built with both intermediate and low level GCMS-FTIR spectral data demonstrated better performance than other models (Table 1). Surprisingly, no significant improvement in terms of R^2^_pred_, SEP, A_*f*_ and B_*f*_ values was observed due to data fusion when compared with models built with only FTIR first derivative data. Models built with FTIR first derivative data demonstrated SEP, A_*f*_ and B_*f*_ of 0.69, 1.08, 0.94 with values of these attributes changing to 0.78, 1.11 and 1.01, respectively for LL-GCMS-FTIR first derivative data fusion. FTIR first derivative data which gave best prediction for models built employing single technique could predict 87 and 74% samples within ±1 Log CFU/g for TVC and Y&M counts, respectively. In contrast, employing data fusion (LL-GCMS-FTIR first derivative) approach, it was possible to predict 87% and 80% samples within ±1 Log CFU/g for TVC and Y&M counts, respectively. Thus it could be clearly concluded that data fusion of these two techniques did not lead to enhanced accuracy or lower bias in the predicted counts. There are several previous reports which suggest improved model performance due to data fusion. But in present study data fusion could not result in better prediction. This might be due to the fact that different techniques used generated data with huge difference in number of variables. In GCMS data only six variables were utilized while FTIR data had 147 variables. Thus, predominance of large matrix of FTIR over GCMS may have reduced efficiency of data fusion^11^.

## Conclusions

Present work attempts to develop chemometric based methods for rapid determination of microbial quality which can probably replace time consuming and labor intensive existing microbial analysis methods. Prediction models were prepared correlating data from FTIR and GCMS with TVC and Y&M employing multivariate statistical tools such as PCA and PLS-R. Models prepared were tested for efficiency by prediction of microbial quality of samples from different batch kept under non-isothermal conditions to simulate market conditions. Results indicate that models built using FTIR data provided good prediction with low SEP and high accuracy. However, prediction results could not be significantly improved by using data fusion techniques. Model built by LL-FTIR first derivative-GCMS data fusion demonstrated similar prediction performance as models built with FTIR. These results suggest possibility of using FTIR for rapid prediction of microbial quality. Furthermore, simple sample preparation and rapid data acquisition time as compared to GCMS are other advantages associated with FTIR instrumentation.

## Methods

### Preparation and withdrawal of sample

Ripe pineapples (*Ananas comosus*) were procured from local market in Mumbai. Pineapples were peeled and cut into thin slices (thickness, 0.2 cm) and mixed to ensure random and homogenous packaging. 75 g of pineapple slices were packaged in polystyrene trays (9 cm × 9 cm × 2.5 cm) and the trays were overwrapped all over using the cling film (Flexo film wraps Ltd. Aurangabad, Maharashtra, India) and then stored at 4 and 10 °C. Samples stored at 4 °C were randomly withdrawn (n = 5) every third day till 22 days, whereas withdrawal of 10 °C stored samples was done every day from day 0 to day 7 (n = 5). A different set of pineapple samples were stored under non-isothermal conditions with periodic 24 h cycle of 16 h at 10 °C and 4 h at 15 °C then finally for 4 h at 20 °C in high precision (±0.5 °C) incubation chambers (MIR-153, Sanyo Electric Co., Osaka, Japan) to simulate possible market conditions. Sample withdrawal for non-isothermal conditions was performed daily in replicates for day 0 to day 4 (n = 3). This set was kept as unknown to check the performance of models.

### Microbial analysis

A portion of 25 g of aseptically cut pineapple pieces were transferred in stomacher bags (Seward, UK) in laminar hood to which 225 ml of 0.9% saline was added. The sample was then homogenized (230 rpm, 1 min) in stomacher (Model: 400 circulator, Seward, UK) and was serially diluted in 0.9% saline. 0.1 mL of appropriate dilution was spread plated in plate count agar (PCA) for total viable aerobic bacterial counts (TVC) and potato dextrose agar (PDA) for total yeast and mould count (Y&M). PCA plates were incubated at 37 °C for 48 h, while PDA plates were incubated at 28 °C for 4 days. Results of the microbial counts were expressed as log_10_ CFU g^−1^.

### Headspace gas chromatography and mass spectrometric analysis (HS-GCMS)

Pineapple cut into pieces (30 g) were added with 7 ml distilled water and then homogenized in omni mixer (Sorvall, Waterbury, CT) for 3 min at Speed 2.5. Resultant slurry was strained through muslin cloth and then centrifuged at 12,850 *g* for 10 min at 4 °C. 15 mL of the juice was added in SPME vial containing 4.5 g of NaCl. 3-hexen-1-ol at final concentration of 28 ug/L was used as internal standard. Headspace volatile compounds were isolated using a pre-conditioned (250 °C, 5 min) solid phase micro extraction (SPME) fiber (50/30 µm polydimethylsiloxane (PDMS)/carboxen (CAR)/divinyl benzene (DVB), Supelco, Bellefonte, PA). Conditions of extraction were: sample equilibration; 40 °C for 10 min with magnetic stirring, fibre exposure for absorption of volatiles; 10 min at same conditions, desorption; on the injection port kept at 250 °C for 2 min. The length of the fibre in the headspace was always kept constant. Before each analysis, the fibre was preconditioned to remove any volatile contaminant by exposing on the injector port for 10 min. Analysis was carried out on GC/MS (QP2020, Shimadzu Corporation, Japan) equipped with a Rxi-5ms capillary column (length = 10 m, inner diameter = 0.1 mm, film thickness = 0.1 µm, Restek Corporation, USA). Helium was used as a carrier gas at a constant flow of 0.4 ml/min. The injector port was equipped with a liner (0.75 mm ID, supelco) suitable for SPME analysis. Injections were conducted in split mode with a split ratio of 5 and GC temperature settings were: Initial oven temperature was 40 °C with a hold time of 5 min. The oven temperature was then increased to 200 °C with change in rate of 13 °C per minute. Oven temperature was finally increased to 280 °C at the rate of 33 °C per minute. Oven was maintained at final temperature for 3 min. The interface temperature was set at 280 °C. MS parameters were: ionization voltage 70 ev, electron multiplier voltage, 1 kV and scan mode from m/z 35 to 500. The peaks were identified by comparing the Kovat indices based on a homologous series of n-alkanes (C5-C24, Aldrich chemical company, WI, USA) with that of standard compounds as well as from MS data available in the Wiley and NIST library^4^ (NIST/EPA/NIH, 2014 compilation). Automated mass detection and identification (AMDIS) software (v 2.62) was used for identification and quantification of target compounds with match factor 90. The peak areas of the targeted volatile compounds were evaluated and quantified based on internal standard to generate a data matrix of observations (sample) and volatile compounds.

### Fourier transform infrared spectroscopy analysis

FTIR spectra was obtained by placing the juice sample (as prepared in section 2.2) on a ZnSe 45°ATR (Attenuated Total Reflectance) crystal of the FTIR spectrometer (Jasco, 4100) equipped with a DLaTGS (deuterated l-alanine doped triglycene sulphate) detector with KBr beam splitter. The spectrometer was controlled by Jasco spectra manager version 2 software. Spectra were collected (average of 40 scans) in the range of wave number 4000–650 cm^−1^ (Fig. S2, See Supplementary Information) with a resolution of 4 cm^−1^. Sample analysis was done in duplicate and mean values of measurements was later used. Background scans were obtained from the blank crystal surface cleaned with distilled water and dried with lint free tissue before each sample analysis to avoid any contaminating peaks^5^.

### Data preparation and data fusion methodologies

GCMS data was arranged as a matrix containing sample wise peak concentrations of identified volatile compound. This matrix was concatenated with their corresponding TVC and Y&M counts. FTIR spectral data in range of 2000 to 1000 cm^−1^ was used for data analysis. Spectra collected was baseline corrected and smoothed using the Savitzky-Golay algorithm of Spectra Manager software (Jasco, Japan). FTIR spectral data was obtained by exporting the ASC II data of spectra to MS Excel. The first derivative data of FTIR spectra was obtained by performing first derivative of FTIR spectral data using a fourth order polynomial with five points by Savitsky-Golay (SG) procedure, ASC II data was then exported to MS excel to obtain first derivative FTIR data. Number of variables obtained for both FTIR spectral and FTIR first derivative data were 1029 (wavenumbers), which were averaged at every seventh point to obtain 147 variables, these variables were arranged sample wise in matrix concatenated with their corresponding TVC and Y&M counts^4^.

Data fusion of data obtained from individual technique such as GCMS with FTIR spectral and FTIR first derivative data was performed. First level data fusion also known as low level data fusion (LL) involves using original data of various measurement methods for model building. It was performed by concatenating data from the two techniques sample wise to form a matrix containing all the information to obtain low level fusion data. Variables of GCMS data with variables of FTIR spectral data and FTIR first derivative data was fused separately. Intermediate level (IL) data fusion invokes data fusion after feature extraction such that to remove dimensionality of data and more so often done by using principal components obtained from PCA or Latent variables obtained from PLS-DA. IL data fusion was performed by obtaining principal components (PCs) of fused data of low level data fusion performing PCA analysis. Number of PCs that explained 95% of cumulative variability of fused data were utilised. The scores of the PCs were then arranged sample wise to obtain the matrix and concatenated with microbial counts. In total, seven categories were considered for model building mentioned as GCMS, FTIR spectral, FTIR first derivative, Low level (LL)-GCMS-FTIR spectral, LL- GCMS-FTIR first derivative, Intermediate level (IL)-GCMS-FTIR spectral and IL-GCMS-FTIR first derivative data. Data was mean centered and standardized before subsequent statistical analysis. All these instrumental observations were treated as independent variable while TVC and Y&M were treated as dependent variables during building of quantitative prediction models

### Mathematical model building and performance evaluation

Principal component analysis (PCA) was performed on GCMS and FTIR data to obtain a visual overview of day wise segregation and clustering of stored packaged pineapple samples. Grubb’s test was performed for outlier detection amongst the samples. The outliers were removed from further chemometric analysis.

Linear regression models for quantitative evaluation of TVC and Y&M were built using partial least square regression (PLS-R) in Chemoface v 1.63 (Brasil). Training data was employed for building and training the model obtained from samples stored at isothermal conditions, 40 [8 (No. of storage days; 0 to 7d) × 5 (No. of samples withdrawn each day)] samples stored at 10 °C was used as training set while unknown set, 25 samples [5 (No. of storage days; 0 to 4 d) × 5 (No. of samples withdrawn each day)] kept under non-isothermal condition was treated as prediction set used for determining performance of the model. A series of PLS models were generated using a number of latent variables ranging from 1 to 21. The performance of each generated model was calculated using leave-one-out cross validation. The performance of PLS models generated were evaluated based on Root mean square error of cross validation (RMSECV). The optimum number of latent variable with lowest RMSECV was then used to build the final model which did not led to overfitting of training subset. The performance parameters for the prediction set subset was then evaluated.

Statistical parameters evaluated for prediction set were Standard error of prediction (SEP), correlation coefficient (R^2^_pred_) accuracy factor (A_*f*_) and bias factor (B_*f*_) between the observed and predicted counts were calculated. The SEP, bias and accuracy factors^34^ were expressed as follows:

1 $${B}_{f}={10}^{(\sum \log ({y}_{i}/y)/n)}$$

2 $${A}_{f}={10}^{({\sum }^{}|log({y}_{i}/y)|/n)}$$

3 $$SEP=\left(\sqrt{\frac{\sum {({y}_{i}-y)}^{2}}{n}}\right)$$

where y_*i*_ is the predicted value of the ith observation, y is the measured value of the ith observation, and n is the number of observations. A_*f*_ explains the degree of average deviation between the observed value and the predicted value from the model. while B_*f*_ explains the directional nature of the predicted count obtained from the model and gives a measure of systematic under or over prediction.

Thus the performance indices for the models build using individual instrument data of GCMS and FTIR was evaluated and low level and intermediate level data fusion was also carried out for both instrument techniques for prediction of TVC and Y&M counts.

## Supplementary information

Supplementary Information.
