# Supplementary material for: Microbial quality assessment of minimally processed pineapple using GCMS and FTIR in tandem with chemometrics
Source: Sci Rep. 2020 Apr 10;10:6203. doi: 10.1038/s41598-020-62895-y (PMC7148306; doi:10.1038/s41598-020-62895-y)

**Microbial quality assessment of minimally processed pineapple using GCMS and FTIR in tandem with chemometrics**

Vanshika Adiani^1,2,#^, Sumit Gupta^1,#^, Prasad S. Variyar^1,2^ *

^1^Food Technology Division, Bhabha Atomic Research Centre,

^2^Homi Bhabha National Institute, Anushakti Nagar,

Mumbai, India

Table S1: Compound table

| Compound | KI Cal | | KI observed | | Day 0 | Day 22 (For 4 °C) | Day 7 (For 10 °C) |
| --- | --- | --- | --- | --- | --- | --- | --- |
| ethanol | | 498.13 | |  | 6.96 ± 0.79 | 6.80 ± 0.81 | 130 ± 12.57 |
| methyl acetate | | 545.96 | |  | 0.00 | 48.48 ± 2.85 | 125 ± 11.38 |
| ethyl acetate | | 616.63 | | 604 | 12.59 ± 1.54 | 106.06 ± 7.01 | 1636 ± 123.12 |
| n propyl acetate | | 704.44 | | 716 | 0.05 ± 0.01 | 0.74 ± 0.08 | 15.45 ± 1.94 |
| methyl butanoate | | 710.89 | | 739 | 6.18 ± 1.07 | 1.02 ± 0.64 | 4.61 ± 0.45 |
| 3-methyl, 1-butanol | | 723.70 | |  | 0.57 ± 0.03 | 0.64 ± 0.08 | 9.34 ± 1.75 |
| methyl,2-methyl butanoate | | 770.26 | | 776 | 11.87 ± 0.82 | 3.30 ± 0.88 | 50.51 ± 6.28 |
| hexanal | | 801.42 | | 804 | 1.06 ± 0.02 | 3.86 ± 0.92 | 0.50 ± 0.07 |
| ethyl butanoate | | 803.34 | | 805 | 0.00 | 1.28 ± 0.07 | 82.39 ± 3.42 |
| Isobutyl acetate | |  | |  | 2.16 ± 0.25 | 0.42 ± 0.06 | 0.00 |
| ethyl 2-methylbutanoate | | 841.75 | | 853 | 2.30 ± 0.36 | 3.60 ± 1.23 | 82.39 ± 3.44 |
| 1-hexanol | | 865.61 | | 860 | 1.90 ± 0.04 | 3.56 ± 0.12 | 3.06 ± 1.60 |
| 1-butanol, 3-methyl acetate | | 871.64 | | 872 | 0.17 ± 0.04 | 0.24 ± 0.03 | 114.82 ± 24.90 |
| 1-butanol, 2-methyl acetate | | 874.75 | | 877 | 3.13 ± 0.77 | 0.11 ± 0.05 | 30.59 ± 4.68 |
| 2- heptanone | | 888.29 | | 889 | 0.108 ± 0.01 | 0.207 ± 0.05 | 18.32 ± 2.04 |
| 2-heptanol | | 901.59 | | 896 | 0.00 | 0.239 ± 0.02 | 9.59 ± 2.24 |
| α thujene | | 929.89 | |  | 11.59 ± 1.49 | 3.14 ± 0.42 | 4.94 ± 0.58 |
| Methyl hexanoate | | 934.82 | | 927 | 107.96 ± 2.58 | 3.04 ± 0.58 | 63.88 ± 4.67 |
| Methyl 3-hexenoate | | 943.53 | |  | 0.31 ± 0.08 | 0.58 ± 0.15 | 2.21 ± 1.08 |
| α sabinene | | 975.25 | | 971 | 97.91 ± 2.04 | 20.36 ± 0.61 | 44.29 ± 6.43 |
| Compound | | **KI Cal** | | **KI observed** | **Day 0**  **(µg/ Kg)** | **Day 22 (For 4 °C)** | **Day 7 (For 10 °C)** |
| 1-heptanol | | 979.95 | | 967 | 0.00 | 2.15 ± 0.67 | 2.26 ± 0.36 |
| ethyl-3-hexenoate | | 1009.02 | | 1002 | 0.19 ± 0.07 | 0.63 ± 0.05 | 7.39 ± 2.99 |
| (+)-4-carene | | 1013.53 | | 1003 | 6.22 ± 0.67 | 1.23 ± 0.22 | 2.16 ± 0.96 |
| o-cymene | | 1022.55 | | 1028 | 4.84 ± 1.18 | 25.20 ± 1.25 | 7.04 ± 2.20 |
| D- Limonene | | 1026.89 | | 1033 | 65.41 ± 3.20 | 25.20 ± 0.66 | 84.20 ± 8.58 |
| 3-(methylthio) methyl propanoate | | 1029.78 | | 1026 | 182.98 ± 17.13 | 26.52 ± 3.70 | 69.45 ± 5.29 |
| 2-ethyl-1-hexanol | | 1033.74 | | 1028 | 14.17 ± 4.73 | 15.45 ± 2.61 | 21.98 ± 3.05 |
| γ terpinene | | 1057.01 | | 1062 | 9.26 ± 2.57 | 2.47 ± 0.86 | 5.75 ± 1.51 |
| Acetophenone | | 1063.97 | | 1078 | 0.45 ± 0.07 | 1.45 ± 0.08 | 2.57 ± 1.01 |
| Dimethyl malonate | | 1074.83 | | 1069 | 0.40 ± 0.27 | 0.79 ± 0.05 | 6.86 ± 2.88 |
| 2,3-butanediol,diacetate | | 1077.70 | | 1080 | 0.72 ± 0.029 | 1.81 ± 0.71 | 16.65 ± 4.9 |
| 3-(methylthio) ethyl propanoate | | 1097.38 | | 1098 | 57.11 ± 1.01 | 26.52 ± 1.70 | 428.04 ± 169.39 |
| Phenyl ethanol | | 1109.17 | | 1120 | 0.00 | 0.07 ± 0.01 | 41.95 ± 4.44 |
| methyl octanoate | | 1121.89 | | 1112 | 107.51 ± 2.32 | 97.61 ± 0.45 | 74.37 ± 16.41 |
| Menthol | | 1166.01 | | 1188 | 18.51 ± 3.09 | 10.21 ± 2.43 | 62.76 ± 30.65 |
| L-4-terpineol | | 1169.77 | | 1188 | 55.16 ± 8.02 | 1.36 ± 0.54 | 3.65 ± 1.43 |
| L-α-terpineol | | 1182.55 | | 1193 | 1.45 ± 0.27 | 5.47 ± 1.08 | 20.81 ± 4.89 |
| Benzenemethanol, .alpha.-methyl-, acetate | | 1186.51 | | 1194 | 1.21 ± 0.07 | 5.16 ± 0.01 | 20.79 ± 7.82 |
| ethyl octanoate | | 1189.43 | | 1196 | 0.26 ± 0.12 | 0.55 ±0.06 | 55.56 ± 12.82 |
| 2-phenylethyl acetate | | 1252.11 | | 1246 | 0.46 ± 0.60 | 0.43 ± | 3.72 ± 0.85 |

Figure S1: GCMS profile at 4 °C (A) & 10 °C (B)


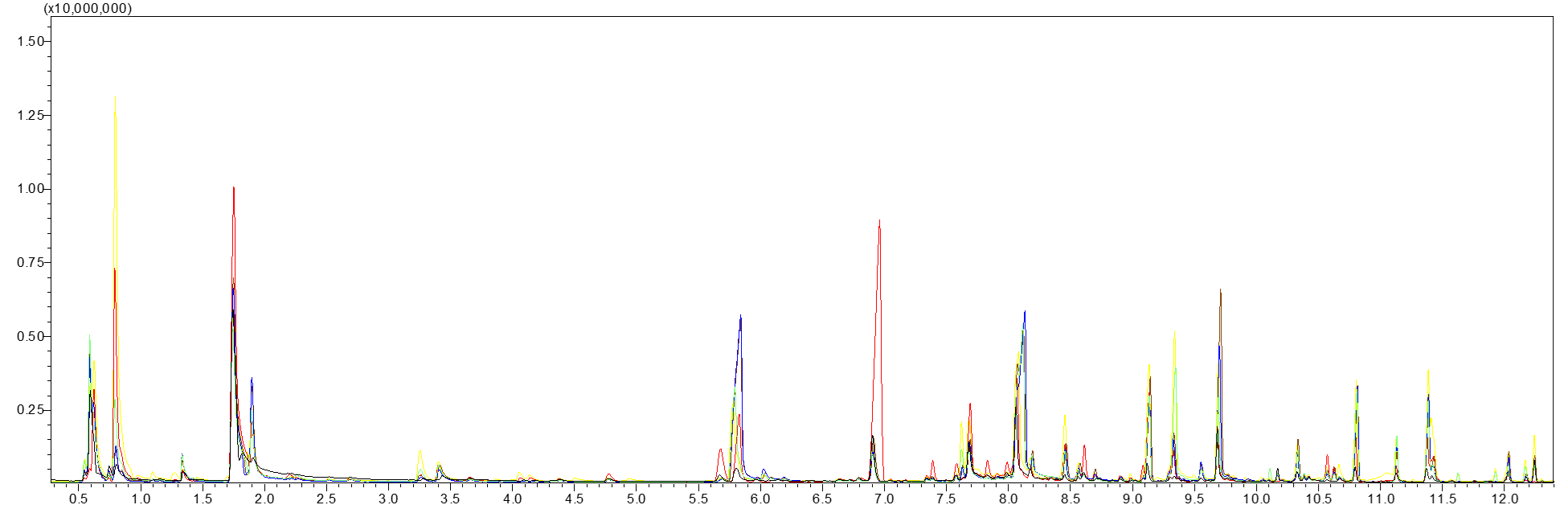


A

Day 0 – Black

Day 3 – Green

Day 6 – Blue

Day 8- Brown

Day 13-Red

Day 20 -Yellow


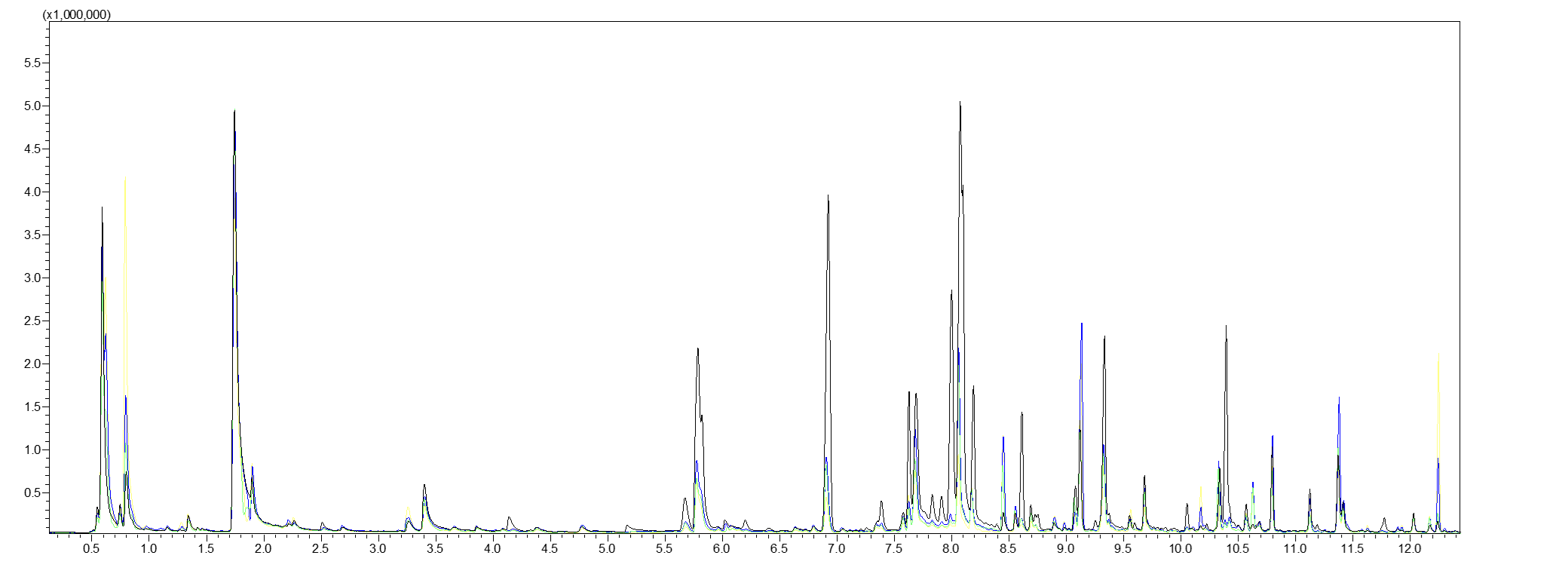

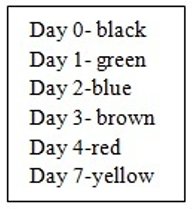


B

Figure S2: FTIR (A) and FTIR first derivative spectral (B) profile at 4 °C


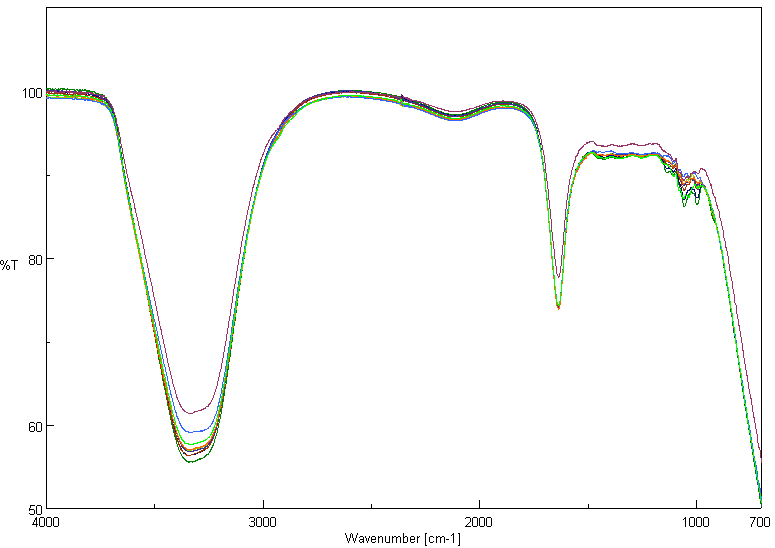

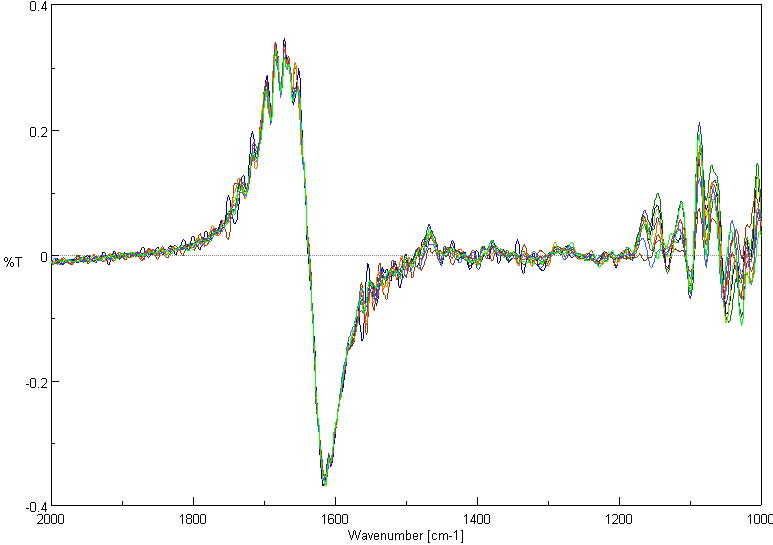


A

B


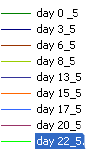

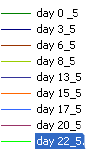


day 22_5

Figure S3: FTIR (A) and FTIR first derivative spectral (B) profile at 10 °C


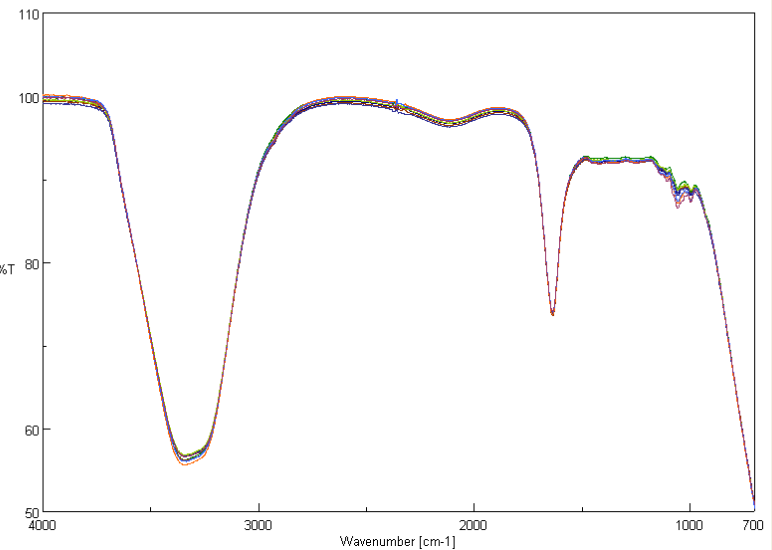

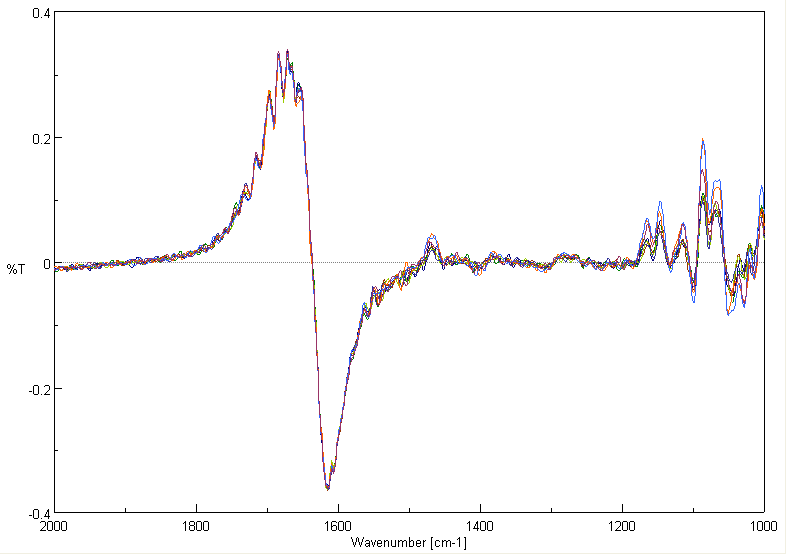

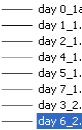

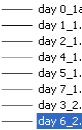


day 6_1

B

A

Figure S4. The plot of LV versus RMSECv during the model building process for all the forms of data. GCMS data, (A & B); FTIR spectral data (C & D); First derivative data (E & F); LL-GCMS-FTIR spectral data (G & H); LL-GCMS-FTIR first derivative data (I & J); IL-GCMS-FTIR spectral data (K & L); IL-GCMS-FTIR first derivative data (M & N). For TVC (A, C, E, G, I, K); for Y&M (B, D, F, H, J, L, M).


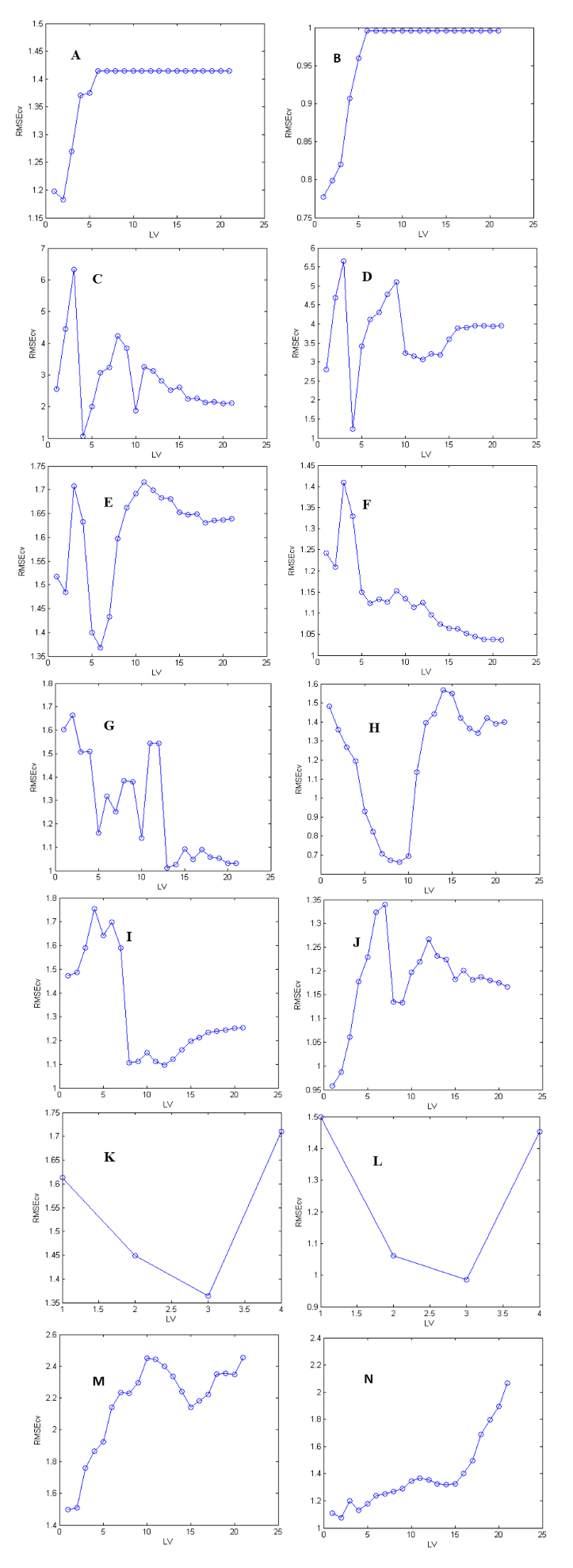

Supplement: Supplementary file 1 — Supplementary Information. [file 41598_2020_62895_MOESM1_ESM.docx]
